# Advancements in the application and research of baculovirus vector vaccines for respiratory diseases in human

**DOI:** 10.3389/fmicb.2025.1558482

**Published:** 2025-03-13

**Authors:** Jinghua Yuan, Jingyu Chen, Qingzhi Zhao, Jialu Xu, Xianwei Li, Yijie Zhang, Hairun Li, Xintong Chen, Ling Zhao, Xiaofen Zhang, Hongyu Li, Keda Chen

**Affiliations:** Laboratory of Artificial Organs and Computational Medicine in Zhejiang Province, Shulan International Medical College, Zhejiang Shuren University, Hangzhou, Zhejiang, China

**Keywords:** baculovirus, vaccine, respiratory disease, preclinical study, clinical application

## Abstract

The rapid spread of respiratory diseases, such as influenza, Severe Acute Respiratory Syndrome Coronavirus 2 (SARS-CoV-2), Middle East Respiratory Syndrome Coronavirus (MERS-CoV), and Respiratory Syncytial Virus (RSV), poses significant challenges to global public health systems. Vaccination remains the most effective strategy to mitigate these threats. Baculovirus Expression Vector Systems (BEVS) have emerged as a promising platform for vaccine development, addressing key limitations of traditional methods, including complex production processes, lengthy timelines, and high costs. BEVS offers distinct advantages, such as enhanced efficacy, safety, cost-effectiveness, and scalability for large-scale manufacturing. This review highlights the application of BEVS in combating respiratory diseases by analyzing preclinical studies, clinical trials, and approved vaccines targeting these pathogens. It also examines recent advancements in BEVS technology, emphasizing its capacity to accelerate vaccine development and respond to emerging respiratory threats. By focusing on the synergy between BEVS and respiratory disease prevention, this review provides valuable insights to guide global vaccine innovation.

## 1 Introduction

Respiratory infectious diseases are highly prevalent and caused by various pathogens, including bacteria, viruses, and other etiological agents. Among these, viruses are the leading contributors to respiratory infections and are transmitted through diverse routes such as airborne, waterborne, foodborne, and direct contact. These pathogens are characterized by high transmissibility, enabling rapid spread and the potential to trigger widespread pandemics. Vaccination plays a pivotal role in preventing viral respiratory infections and mitigating the risk of epidemics.

We searched the official WHO website for systematic data on the epidemiology of respiratory diseases and conducted comprehensive searches in PubMed, ClinicalTrials.gov, and EMA databases to collect references to 1,362 relevant articles covering the period to February 21, 2025 with keywords about baculovirus, vaccine, respiratory disease, preclinical study, clinical application. We read and excluded dissertations, presentations, gray literature, non-English language papers, extended abstracts, and duplicate publications. Ultimately, 136 records were validly referenced. In this narrative review, we outline the current epidemiology of several common or hazardous respiratory infectious diseases, summarize the fundamentals of the Baculovirus Expression Vector System (BEVS), and analyze baculovirus-vectored vaccines in comparison to conventional vaccine platforms. In particular, we focused on the development and application of baculovirus vector vaccines for respiratory infectious diseases. Through rigorous screening of information, we successfully compiled the latest experimental evidence covering preclinical studies and clinical trials, as well as summarizing the currently approved baculovirus vector vaccine products.

### 1.1 Epidemic status and conventional vaccine research of respiratory diseases

In the past 6 years, global pandemics have been caused by viruses such as influenza, Respiratory Syncytial Virus (RSV), and Severe Acute Respiratory Syndrome Coronavirus 2 (SARS-CoV-2). Additionally, localized outbreaks of Middle East Respiratory Syndrome Coronavirus (MERS-CoV) have been reported in Saudi Arabia since 2012 (WHO, 2022). According to the WHO the influenza virus infects over 1 billion individuals annually, with 3–5 million severe cases and 290,000–650,000 respiratory-related deaths (WHO, 2023a). In 2019, more than 30 million RSV infections were reported in children under 5 years old (Venkatesan, 2024). As of March 11, 2023, Corona Virus Disease 2019 (COVID-19) has resulted in over 759 million confirmed cases and 6.9 million deaths globally (WHO, 2023b). Furthermore, since April 2012, MERS-CoV has caused 2,609 documented cases worldwide, with a mortality rate of 36% (MERS Situation Update, 2023). The global economic burden of respiratory viral diseases, including medical costs, productivity losses, and vaccine investments, amounts to billions of dollars annually. The recurrent emergence of these diseases continues to pose a significant challenge to global public health (Rodrigues and Plotkin, 2020). By inducing herd immunity, vaccination significantly reduces the incidence and fatality rates of these infections. Currently, vaccines for viral respiratory diseases predominantly include inactivated and live attenuated formulations.

**Figure 1:**
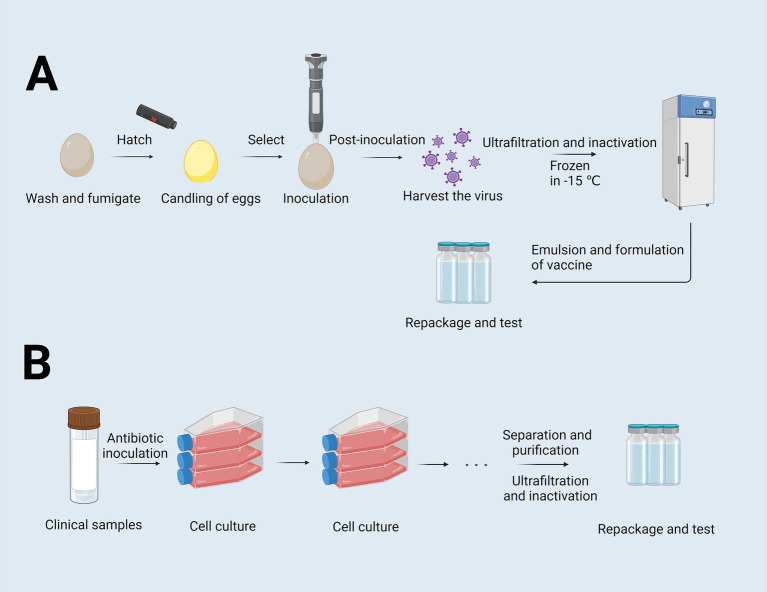
Flowchart of the egg-based and cell-culture-based vaccine production process. **(A)** Flowchart of egg-based inactivated vaccines. Eggs were washed and fumigated, and then were selected by examining the internal structure of the eggs through an egg candling lamp to exclude defective eggs. Following inoculation, the virus is harvested, purified, and inactivated to render it suitable for vaccine use. The virus is then ultrafiltered, further inactivated, and cryopreserved at −15°C. Subsequent steps involve the antigen is emulsified with adjuvants, formulated into doses, repackaged and tested. **(B)** Flowchart of cell-culture-based culture-inactivated vaccines. This process starts with clinical sample collection, followed by cell inoculation and expansion. After sufficient cell growth, the virus is harvested, purified, ultrafiltered, and inactivated. Finally, the inactivated virus is formulated, repackaged, and subjected to quality control examinations to ensure safety and efficacy (Zulkarnain et al., 2021). Created with BioRender.com.

The production of inactivated vaccines primarily relies on two conventional approaches: the egg-based inactivated vaccine technique (Figure 1) and the cell-culture-based inactivated vaccine technique (Figure 1). Although the chicken embryo culture technique, introduced in the 1950s, has achieved substantial advancements, it is constrained by several limitations. These include the dependence on specific pathogen-free (SPF) chicken embryo cells, lengthy production timelines, complex procedures, and susceptibility to external factors. Such constraints hinder its ability to meet the growing vaccine demand, particularly during outbreaks of highly pathogenic respiratory diseases (Wong and Webby, 2013). In contrast, the cell culture-based production of inactivated vaccines offers several advantages, including simplified processes, improved storage and transportation capabilities, enhanced development of combination vaccines, and high safety profiles. Nevertheless, this technique faces persistent challenges, such as viral antigenic drift, limited vaccine efficacy, and the requirement for high-dose formulations to achieve adequate protection (Wong and Webby, 2013). Live attenuated vaccines, on the other hand, retain immunogenicity while reducing toxicity, making them widely applicable. However, the residual pathogen activity in these vaccines presents a potential risk of infection, posing safety concerns and complicating their development and deployment.

The emergence of new viral infectious diseases has spurred significant advancements in traditional vaccine technologies and has prompted the exploration of innovative approaches to vaccine production. Recombinant vaccines, produced through genetic engineering techniques, are gaining increasing popularity in the field of vaccine research and development. These vaccines offer an advantage by avoiding the safety risks associated with traditional vaccines that use live viruses or bacteria. In 1986, the U.S. Food and Drug Administration (FDA) approved the first recombinant DNA vaccine for Hepatitis B, marking a pivotal milestone in global efforts to control and prevent Hepatitis B outbreaks. This achievement represented a major step forward in recombinant vaccine development and its clinical application (Venters et al., 2004).

The conventional recombinant vaccine production process involves introducing a target gene into a vector using genetic engineering techniques, followed by transfer into host cells for protein expression and production. While this method provides high safety levels and a relatively straightforward production process, it is associated with substantial costs, lengthy production timelines, and limited yields. These factors hinder the ability to rapidly produce vaccines against diverse pathogens.

### 1.2 Development and characteristics of baculovirus expression vector systems

Baculoviruses are insect-specific DNA viruses (Ferrelli and Salvador, 2023). Among the common viral vectors, baculoviruses are unique in their ability to accommodate large amounts of heterologous DNA and accurately deliver this material to selected host cells (Gupta et al., 2019). Therefore, recombinant baculoviruses have many applications, the best known of which are large-scale protein production systems in combination with insect cell culture (Rodríguez-Hernández et al., 2023). In the 1980s, BEVS emerged as a prominent technology for expressing exogenous proteins. BEVS offered an optimized platform that overcame many of the limitations of conventional recombinant vaccine production. Following over a decade of development, BEVS technology led to the introduction of the first commercially available vaccine for cervical cancer in 2007 (EMA, 2007; Hong et al., 2023). This vaccine not only addressed the prolonged production cycles inherent in traditional recombinant vaccines but also demonstrated the high efficiency of BEVS in producing complex proteins. The BEVS platform has since become a leading technology for the production of viral vaccines and gene therapy vectors, providing a rapid, cost-effective, and highly efficient approach to vaccine development, thus significantly advancing the field of vaccinology and therapeutic applications (Yoshida et al., 2010; Yoshida et al., 2003; Yang et al., 2007; Tao et al., 2009; Kolpe et al., 2012; Chen et al., 2010).

**Figure 2:**
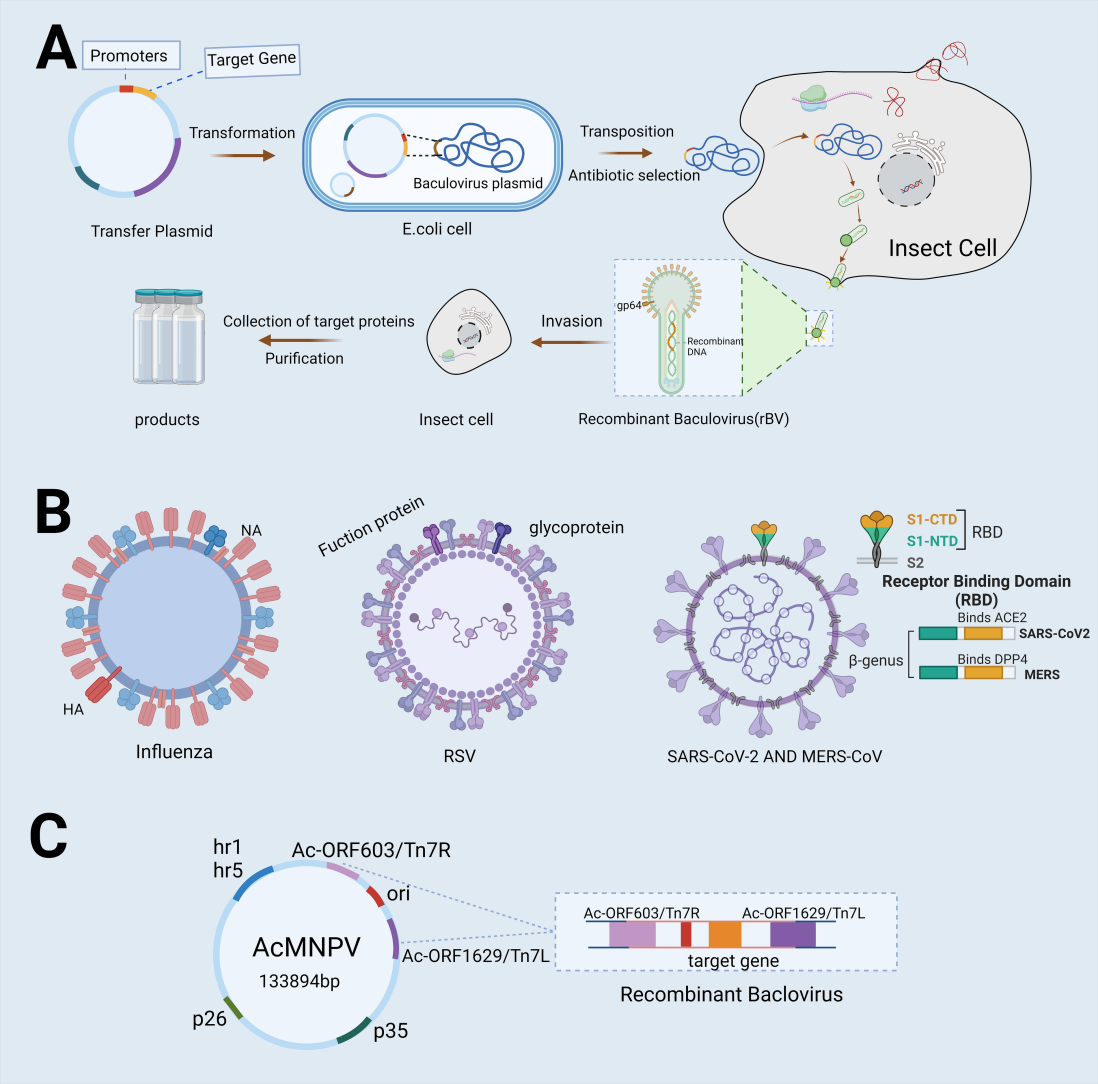
Flowchart depicting the utilization of Bac-to-Bac for vaccine development. **(A)** The flowchart illustrates the production process of Baculovirus Expression Vector Systems (BEVS) utilizing the advanced Bac-to-Bac technology. The target gene is first integrated into a transfer plasmid, which is then introduced into *Escherichia coli* harboring the baculovirus plasmid. This facilitates partial homologous recombination between the transfer plasmid and the baculovirus plasmid, resulting in the production of recombinant baculovirus DNA. Once introduced into insect cells, this recombinant DNA triggers the production of high concentrations of recombinant baculovirus particles. The virus then infects the cells, leading to the collection and purification of proteins for the production of a recombinant protein vaccine. **(B)** Target genes used in this system include the hemagglutinin (HA) and neuraminidase (NA) genes of influenza, the fusion protein and glycoprotein genes of Respiratory Syncytial Virus (RSV), and the Spike Receptor Binding Domain (S-RBD) structures of Severe Acute Respiratory Syndrome Coronavirus 2(SARS-CoV-2) and Middle East Respiratory Syndrome Coronavirus (MERS-CoV). **(C)** The most commonly used baculovirus vectors include *Autographa californica multiple enveloped nuclear polyhedrosis virus (AcMNPV)*, which is capable of replicating within *Escherichia coli* and exhibits infectivity toward lepidopteran cells. Created with BioRender.com.

The development of baculovirus vector vaccines typically employs the Bac-to-Bac system, which consists of five primary stages: (1) The target gene is integrated into a transfer plasmid; (2) Recombination occurs between the target gene and the baculovirus plasmid, resulting in the formation of recombinant baculovirus DNA; (3) The recombinant baculovirus DNA is introduced into insect cells, where it undergoes transcription and subsequent encapsulation to produce recombinant baculovirus particles; (4) The recombinant baculovirus is harvested and used to infect new insect cells, generating a large quantity of the target protein (Felberbaum, 2015) (Figure 2A).

### 1.3 Advantages of the baculovirus vector vaccine over conventional vaccines

The utilization of BEVS in vaccine development offers several advantages over conventional methods: (1) High Safety: Baculoviruses activated in mammalian cells rely on the specific mammalian gene promoters (Hofmann et al., 1995). These viruses cannot replicate within mammalian cells, nor do they integrate the target gene into the chromosomal DNA of mammals (Chen et al., 2011; Lu et al., 2012). As a result, they do not pose a risk of disease in mammals or humans (Chen et al., 2010; Sung et al., 2014). (2) High Flexibility: The baculovirus genome is capable of incorporating multiple genes or large exogenous gene fragments, enabling efficient post-translational modifications. This feature makes it particularly suitable for expressing protein-based vaccines that are complex or challenging to produce using traditional methods (Contreras-Gómez et al., 2014). (3) High Plasticity: Insect cells used in BEVS have the capacity to perform various post-translational modifications on exogenous proteins, including glycosylation, phosphorylation, and acetylation (Drugmand et al., 2012). (4) High Efficiency: The baculovirus genome contains strong promoters such as ph/p10, which drive efficient transcription of exogenous proteins (Rong et al., 2019). However, the immunogenicity of the resulting vaccine is typically modest, as it relies only on partial viral proteins. Therefore, incorporating appropriate adjuvants is necessary to enhance the immune response.

These advantages hold significant potential for advancing vaccines against viral respiratory diseases. Previous reviews have thoroughly examined the research progress, immunogenicity, and protective efficacy of various vaccine types, including inactivated vaccines, live attenuated vaccines, protein subunit vaccines, virus-like particle (VLP) vaccines, mRNA vaccines, and viral vector vaccines (Pollard and Bijker, 2021; Osterholm et al., 2012). This review provides an up-to-date overview of the research progress on baculovirus vector vaccines for common viral respiratory diseases, including clinical trials and approved vaccines listed in Table 1. Additionally, it critically evaluates the sustainability, feasibility, and inherent limitations of BEVS-based vaccines in clinical applications for viral respiratory infections.

**Table 1:** Summary of vaccines in clinical trials and already approved.

	Target	Antigen	Band name	Manufacture	Stage	References
Human respiratory vaccines	Influenza virus	HA protein	FluBlok	Sanofi Pasteur	Approved (STN:125285)	Flublok (2024) and Cox et al. (2008)
	Influenza virus	HA protein	Flublok Quadrivalent	Sanofi Pasteur	Approved (STN:125285)	Dunkle et al. (2017a) and Flublok Quadrivalent (2024)
	COVID-19	Spike (S) protein	Nuvaxovid/Covovax	Novavax	Approved (EMA: EMEA/H/C/005991)	Guebre-Xabier et al. (2020) and Novavax COVID-19 Vaccine (2024)
	COVID-19	recombinant RBD monomer	Coviccine	WestVac Biopharma/ West China Hospital of Sichuan University	Approved	Yang et al. (2020) and Biopharma (2024)
	COVID-19	recombinant RBD monomer	Coviccine® Trivalent XBB.1.5-Recombinant COVID-19 Trivalent (XBB.1.5 + BA.5 + Delta) Protein Vaccine (Sf9 cell)	WestVac Biopharma/ West China Medical Center/West China Hospital of Sichuan University	Approved	Biopharma (2023) and Biopharma (2024)
	COVID-19	CoV2 preS dTM	Sanofi-GSK	Sanofi/GSK	Approved (EMA: EMEA/H/C/005754)	Pavot et al. (2022)
	Influenza A H1N1	A (H1N1) 2009 Influenza Virus-like Particle	—	Novavax	Phase II (Clinical trial No.: NCT:01072799)	López-Macías et al. (2011) and ClinicalTrials (2020)

	Influenza virus	Hemagglutinin (HA), neuraminidase (NA) and matrix 1 (M1)	Nanoflu	Novavax	Phase III (Clinical trial No.: NCT:04120194)	Smith et al. (2017)
	Respiratory syncytial virus	Fusion glycoprotein	Resvax	Novavax	Phase III (Clinical trial No.: NCT 02624947)	Fries et al. (2017) and Jares Baglivo and Polack (2019)
	COVID-19 and Influenza	Quadrivalent Hemagglutinin Nanoparticle Influenza and SARS-CoV-2 rS Nanoparticle	qNIV/CoV2373 vaccine	Novavax	Phase II (Clinical trial No.: NCT 05519839)	Massare et al. (2021) and A Study to Evaluate the Safety and Immunogenicity of COVID-19 and Influenza Combination Vaccine (COVID-19) (2024)

## 2 COVID-19

### 2.1 Epidemiology of COVID-19

In December 2019, COVID-19 emerged in Wuhan, China, and rapidly spread both nationally and internationally, posing a significant threat to public health. SARS-CoV-2, the virus responsible for COVID-19, is a single-stranded RNA virus. Its genome encodes four primary proteins: the spike (S) protein, membrane (M) protein, envelope (E) protein, and nucleocapsid (N) protein. The M protein is a key component of the viral envelope, playing a critical role in maintaining the structural integrity and stability of the virus. The N protein, a structural protein, is primarily involved in viral replication and assembly, facilitating replication within host cells and the formation of new viral particles. The E protein contributes to viral assembly and release and is involved in the fusion and entry of the virus into host cells. The S protein functions as a ligand that binds to the angiotensin-converting enzyme 2 (ACE2) receptor in human cells, enabling the virus to enter host cells. This interaction is essential for SARS-CoV-2 infection. As such, the S protein is a critical immunogen and is widely utilized in vaccine development (Figure 2B).

Over the past 3 years, genetic mutations in the SARS-CoV-2 genome have led to the emergence of new variants, including the Alpha, Beta, Gamma, Delta, Epsilon, and Omicron strains. With the advent of these mutations, the development of SARS-CoV-2-targeting vaccines has advanced rapidly. Current vaccines against SARS-CoV-2 include inactivated virus vaccines, live attenuated vaccines, viral vector vaccines, protein subunit vaccines, RNA vaccines, DNA vaccines, VLP vaccines, mRNA vaccines, and adenovirus-based vaccines. The highly adaptable and flexible manufacturing processes of baculovirus vector vaccines allow for swift modifications to address emerging viral mutations. This capability makes them particularly valuable in the research and development of SARS-CoV-2 vaccines, drawing considerable attention for further exploration (Wong and Webby, 2013; Gebre et al., 2021).

### 2.2 Preclinical investigations

Fujita et al. (2020) developed a rapid method to efficiently construct the extracellular domain of the SARS-CoV-2 S protein using the baculovirus-silkworm expression system, achieving successful purification. Building on this, Smith et al. (2021) employed BEVS to express recombinant S protein in insect larvae, utilizing live larvae rather than traditional cell cultures. The recombinant S protein is secreted into the hemolymph, facilitating easier extraction. This approach provided an efficient and cost-effective platform for large-scale production of the S protein. Furthermore, Smith et al. (2021) established an ELISA method to detect anti-S-specific IgG antibodies in the serum/plasma of COVID-19 patients. The assay demonstrated high sensitivity and specificity, with values reaching 96.2 and 98.8%, respectively, confirming the immunoreactivity of the insect-derived S protein to the sera of COVID-19 patients.

To enhance vaccine efficacy, Cho et al. (2021) employed *Autographa californica multiple nucleopolyhedrovirus (AcMNPV)* as a vector to construct a recombinant baculovirus, AcHERV, capable of expressing the outer membrane glycoprotein from a human endogenous retrovirus (HERV). AcHERV utilizes the type D retroviral receptor on human cells and relies on cellular endocytosis for efficient gene delivery into host cells. The gene encoding the S protein was successfully integrated into AcHERV and designated as acHERV-COVID-19-S. In murine models, immunization with the acHERV-COVID-19-S vaccine induced the production of serum IgG, neutralizing antibodies, and antigen-specific IFN-*γ* secretion, providing resistance against viral infection. These findings underscore the potential of AcHERV as a promising vector for developing vaccines against SARS-CoV-2.

In addition to expressing the S protein, Sullivan et al. (2022) successfully generated VLPs of SARS-CoV-2 by employing BEVS for the co-expression of the S, M, and E proteins. Immunization of Syrian hamsters with this antigen resulted in the production of neutralizing antibodies in all vaccinated animals. Upon viral challenge, antibody production was further enhanced, and no adverse events were recorded in the vaccinated animals (Felberbaum, 2015; Targovnik et al., 2021). This experiment demonstrated the immunogenicity of VLPs produced by BEVS without the need for adjuvants. Although further in-depth studies in animal trials are needed, the successful production of VLPs provides a valuable foundation for the continued research and development of vaccines (Targovnik et al., 2021; van Oosten et al., 2021).

### 2.3 Progress of vaccines in clinical trials and approved vaccines

Sanofi and GSK developed a recombinant protein vaccine utilizing BEVS, which has completed Phase III clinical trials. This vaccine combines Sanofi’s recombinant protein technology, previously employed in influenza vaccines, with GSK’s patented adjuvant technology, AS03, enabling efficient large-scale production of the SARS-CoV-2 S protein. The integration of AS03 not only enhances the vaccine’s immunogenicity but also reduces the required protein dosage per dose, significantly improving vaccine efficacy while mitigating financial costs. The results of the Phase III trial, announced on February 24, 2022, demonstrated that as a booster vaccine, it significantly increased neutralizing antibody levels by 18–30 times in adults and the elderly who had previously received mRNA or adenovirus vaccines, and by 84–153 times in those who had received two doses of the Sanofi-GSK vaccine. Additionally, the trial revealed that the vaccine had an efficacy of 57.9% (95% CI 26.5–76.7) in preventing symptomatic COVID-19 following two doses in a seronegative population. Importantly, none of the vaccinated individuals developed severe symptoms or required hospitalization due to COVID-19, indicating 100% protection against such outcomes. This vaccine has received approval from regulatory authorities such as the FDA and the European Medicines Agency (EMA) and has been widely deployed (Mahase, 2022).

On July 13, 2022, Novavax developed the NVX-CoV2373 recombinant protein vaccine using BEVS. This vaccine targets the S protein of SARS-CoV-2, and is adjuvanted with Novavax’s proprietary Matrix-M to enhance immune responses and elevate neutralizing antibody levels. The NVX-CoV2373 vaccine induces IgG antibodies that inhibit the binding of the S protein from various SARS-CoV-2 variants to the human angiotensin-converting enzyme 2 (hACE2) receptor, thus reducing the likelihood of viral entry into lung, throat, and intestinal cells. In clinical trials involving adolescents aged 12–17, the vaccine demonstrated a disease incidence of 0.5%, compared to 2.4% in the placebo group. These findings suggest an efficacy of approximately 79.2% in this cohort (European Medicines Agency, 2022). In adults aged 18–59, the vaccine elicited a 220-fold increase in neutralizing antibody titers by day 35 post-vaccination, along with a 100% seroconversion rate (Underwood et al., 2023). NVX-CoV2373 has been authorized for use in two key scenarios: emergency use for individuals aged ≥12, and as a booster for adults aged ≥18. The vaccine exhibited acceptable immunogenicity and a favorable safety profile, with minimal adverse events such as tenderness, pain, and swelling at the injection site. The vaccine has received Emergency Use Authorization (EUA) from the FDA for COVID-19 prevention.

The Omicron variant of SARS-CoV-2, first identified in South Africa in November 2021, rapidly spread to various countries and regions within weeks. By December 2022, Omicron emerged as the dominant strain in China. Known for its high transmissibility, strong contagion, and stealthy infectious latency, the Omicron variant presented unique challenges to global public health. In response, WestVac Biopharma, in collaboration with Sichuan University West China, developed Coviccine™, a recombinant vaccine produced using BEVS in Sf9 cells. Approved by Chinese authorities on December 5, 2022, Coviccine™ is the first recombinant SARS-CoV-2 vaccine in China to employ insect cell technology through BEVS. The vaccine incorporates the spike receptor-binding domain (S-RBD) derived from the SARS-CoV-2 spike protein and aluminum hydroxide as an adjuvant (Meng et al., 2021). Coviccine™ has demonstrated promising cross-protection against various variants, including Omicron, and is now being used as a second booster in China’s COVID-19 immunization program. It is particularly recommended for high-risk populations, including individuals aged 60 and above, and those with underlying health conditions (Meng et al., 2021).

In August 2022, the XBB variant of SARS-CoV-2 emerged and rapidly spread worldwide, demonstrating enhanced immune evasion capabilities. As a result, it remains capable of infecting individuals previously infected with other variants, highlighting the urgent need for the development of a targeted XBB vaccine. In response, WestVac Biopharma, in collaboration with Sichuan University West China, initiated the development of a vaccine specifically designed to target the XBB variant. This enhanced version of the Coviccine vaccine, named the Coviccine XBB Vaccine, utilizes BEVS as its platform. The vaccine incorporates a stable trimeric protein particle designed to target the S-RBD and heptad repeat (HR) regions of SARS-CoV-2 variants, including XBB and BA.5. Two weeks post-vaccination, the vaccine demonstrated a robust protective efficacy of 93.28% against symptomatic COVID-19 caused by variants such as XBB.1, XBB.1.5, and XBB.1.9, while maintaining an excellent safety profile. On June 8, 2023, Chinese authorities approved the emergency use of this product, and on June 15, 2023, the FDA granted global emergency use authorization for the first COVID-19 vaccine specifically targeting XBB variants (Biopharma, 2023).

In addition to its use in the development of monovalent vaccines for SARS-CoV-2, BEVS can also facilitate the creation of combination vaccines targeting multiple diseases. Data from Public Health England indicate that co-infections are most prevalent among the elderly, with fatal outcomes occurring in over 50% of cases. This highlights the urgent need for combination vaccines. On December 22, 2021, the World Health Organization granted emergency use listing approval to Novavax’s combination respiratory vaccine (qNIV/CoV2373), which combines a recombinant SARS-CoV-2 spike (S) protein with Matrix-M adjuvant and a quadrivalent seasonal influenza hemagglutinin nanoparticle.

The data demonstrate that the qNIV/CoV2373 combination vaccine not only induces a robust polyclonal antibody response against the original SARS-CoV-2 strain (US-WA) but also elicits a potent polyclonal antibody response targeting the neutralizing epitopes of the receptor-binding domain (RBD) in the B.1.351 South African variant. These findings suggest that the combined vaccine can trigger a cross-reactive immune response, effectively targeting two distinct variants of SARS-CoV-2. Moreover, the vaccine is unique in its ability to generate substantial levels of Hemagglutination Inhibition (HAI) and neutralizing antibodies, which target both influenza A and B strains. The qNIV/CoV2373 vaccine also induces antibodies that block the interaction between the SARS-CoV-2 S protein and the human angiotensin-converting enzyme 2 (hACE2) receptor. This combined vaccine could serve as an effective preventive measure against both seasonal influenza and COVID-19 (Massare et al., 2021).

## 3 Influenza

### 3.1 Epidemiology of influenza

**Figure 3:**
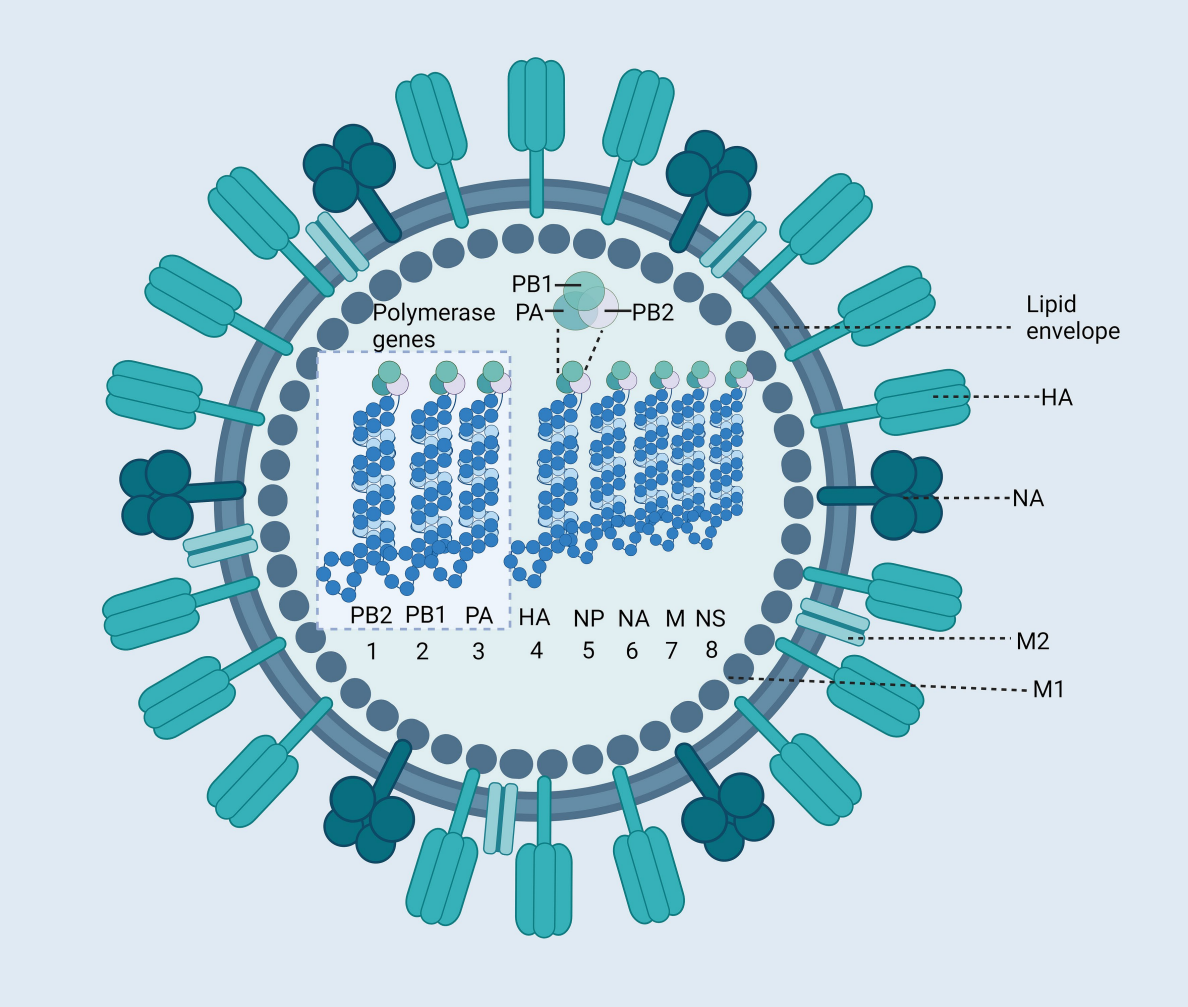
Structure of the Influenza Virus. The influenza virus is an enveloped virus, with two major glycoproteins: hemagglutinin (HA) and neuraminidase (NA). The core of the virus contains the segmented negative-sense RNA genome, which consists of eight segments (WHO, 2022; WHO, 2023a; Venkatesan, 2024; WHO, 2023b; MERS Situation Update, 2023; Rodrigues and Plotkin, 2020; Wong and Webby, 2013; Venters et al., 2004) encapsidated by nucleoproteins (NP). These RNA segments encode various viral proteins, including polymerase basic protein 2 (PB2), polymerase basic protein 1 (PB1), polymerase acidic protein (PA), HA, NP, NA, Matrix protein 1 (M1), Matrix protein 2 (M2), Non-structural protein (NS). Created with BioRender.com.

During annual influenza outbreaks, children exhibit the highest incidence rates, accounting for 20–30% of pediatric cases (Ghebrehewet et al., 2016; Fraaij and Heikkinen, 2011). Vaccines remain the most effective strategy for preventing influenza. The influenza virus is an enveloped, negative-sense single-stranded RNA virus with a segmented genome. Both Influenza A and B viruses possess genomes composed of eight RNA segments that encode several key components: RNA polymerase subunits, viral glycoproteins—including hemagglutinin (HA) with its distinct globular “head” and “stalk” structure, and neuraminidase (NA), which facilitates viral entry—nucleoprotein (NP), matrix protein (M1), membrane protein (M2), non-structural protein NS1, and nuclear export protein (NEP) (Figure 3). The initial step for viral entry into host cells involves the interaction between HA and cellular receptors. NA plays a critical role by enzymatically cleaving the glycoprotein linkages between viral particles and the cell surface, thus enabling the efficient release of newly formed virions for subsequent infection of other cells. Antigenic variation in the influenza virus refers to changes in the structures of HA and NA antigens. Antigenic drift and shift necessitate frequent updates to influenza vaccines to ensure they align with emerging viral strains. Antigenic variation in influenza A viruses typically occurs every 1–3 years, whereas antigenic changes in influenza B viruses are much slower (Krammer et al., 2018). Influenza C and D viruses, which possess only seven RNA segments, do not cause significant pathogenicity in humans. Consequently, this review will focus exclusively on Influenza A and B viruses.

The development of influenza vaccines has a long and rich history, with significant milestones achieved over the years. Notably, the successful cultivation of the influenza virus in chicken embryos dates back to 1936. Currently, influenza vaccines are available in various forms, including inactivated vaccines (such as whole-virus inactivation, split, and subunit vaccines), live attenuated vaccines, recombinant protein vaccines, RNA vaccines, and DNA vaccines. Among these, baculovirus vector vaccines stand out due to their unique advantages in the context of influenza vaccine development (Table 2). These advantages have played a critical role in accelerating the development and clinical progression of baculovirus vector-based influenza vaccines.

**Table 2:** Comparison of baculovirus vector influenza vaccine with different types of influenza vaccines.

Vaccine types	Advantages	Disadvantages	References
Inactivated vaccines (including whole virus inactivation, split, and purified subunit vaccines)	Safe for pregnant women Suitable for immunocompromised patients Adjuvants can enhance the antibody titers and cellular immune responses	May cause injection site pain, fever, headaches, muscle pain General discomfort are mostly observed in children Induce weaker immune responses in young children and the elderly than in adults Long production cycle May cause allergies Erythema may be caused by adjuvants	Jamali et al. (2018), Kim et al. (2022), Zhao et al. (2023), Moro et al. (2020)
Attenuated live vaccine	Systemic allergic reactions such as urticaria, angioedema, rhinitis, eczema, etc. have not yet been seen Can be administered via nasal spray (common in children)	May cause mild to moderate symptoms, including runny nose, sneezing, nasal discomfort, fever, and headache Can cause one or more adverse reactions in children, including arm pain, chills, myalgia Not recommended for routine use in pregnant women	Clements and Murphy (1986), Singanayagam et al. (2018), Tanner et al. (2021), Loeb et al. (2016)
Baculovirus vector vaccine	High security Well tolerated with few side effects Rapid and stable induction of humoral and cellular immune responses	Low doses may reduce immunogenicity Need increased dosage to improve immunogenicity	Felberbaum (2015) and Soema et al. (2015)
RNA vaccine	Safety Efficacy High potency Ability to design multivalent vaccines encoding antigens of known influenza virus subtypes Possibility of mucosal delivery	May cause mild to moderate symptoms, including injection site pain, fever, headache, fatigue, myalgia, arthralgia, nausea, chills Higher costs Adverse effects such as thrombosis and/or edema may occur	Vogel et al. (2018), Arevalo et al. (2022), Liu et al. (2024), Bahl et al. (2017), Soens et al. (2025)
DNA vaccine	Possibility of mucosal delivery Stimulates innate immunity Adjuvants can enhance the antibody titers and cellular immune responses	Low immunogenicity Risk of integration of DNA vaccine genetic material into cellular or host DNA May cause autoimmune disorders against host DNA Mild to moderate flu-like symptoms and injection site reactions that may be caused by adjuvants	Zhao et al. (2023), Loudon et al. (2010), Alexander et al. (2010), Chen et al. (2008), Vollmer and Krieg (2009)
Virus-like particles vaccine	Elicits a strong and persistent immune response Ability to load immunomodulators	Multi-step process Less immunogenicity compared to other platforms	Durous et al. (2019)
Adenovirus vectored vaccines	High expression levels of the transgene of the antigenic protein Ability to simultaneously induce humoral and cellular immune responses Safe, easy to prepare and do not require adjuvants	Innate inflammatory response may lead to systemic toxicity after adenovirus vector administration but the probability is low	Appledorn et al. (2008), Zhu et al. (2007), Ahi et al. (2011)
Virosomes vaccines	Good immunogenicity in both healthy and immunocompromised elderly, adults and children	Without adjuvants, virosomes poorly activate the antigen-presenting cells and fail to trigger cross-presentation, limiting the immunity induction in antigen-presenting cells	Herzog et al. (2009)

### 3.2 Preclinical investigations

The influenza virus is characterized by frequent antigenic drift, necessitating the annual update of vaccine compositions. Existing influenza vaccines, however, offer limited protection and provide immunity for only a short duration. Consequently, there is a pressing need to enhance traditional influenza vaccines and develop a universal vaccine that can reduce disease burden and prevent widespread outbreaks. In the past 2 years, the H3N2 influenza virus has emerged as the dominant global strain. To address this, Liu et al. (2023) Optimized HA and NA antigens of H3N2 and expressed them as chimeric VLPs using the BEVS. They compared the protective efficacy of this chimeric VLP with that of a commercially available quadrivalent inactivated influenza vaccine (QIV). In mouse experiments, a single dose of the chimeric VLP induced robust cross-reactive antibody responses, while the QIV only elicited limited antibody production against non-matching influenza strains. These findings suggest that the chimeric VLP holds promise as a vaccine candidate, with broad cross-reactivity and significant potential for improving influenza vaccine efficacy.

Influenza vaccines are developed not only to target highly epidemic strains but also to address other prevalent influenza viruses. The H7N9 influenza subtype, first identified in humans in March 2013, has become a major concern due to its significant impact on both human health and the poultry industry (Gao et al., 2013). In 2022, Hu et al. (2022) Employed BEVS to develop a promising vaccine candidate against H7N9, incorporating HA, NA, and M1. The efficacy of this vaccine was evaluated in both avian species and murine models. The results demonstrated that intramuscular administration of the H7N9 VLP induced a strong humoral immune response, significantly reduced the expression of key pro-inflammatory factors in murine lung tissue, and effectively suppressed H7N9 viral infection. These positive outcomes in both chicken and mouse models highlight the potential of BEVS-derived VLP vaccines as viable alternatives to conventional egg-based vaccines for combating H7N9 influenza in both humans and poultry. However, the study focused exclusively on animal-level efficacy, and no further advancements have been reported.

In contrast to the HA + NA + M1 VLP vaccine targeting the H7N9 virus, the H5N6 VLP serves as a candidate antigen for a broad-spectrum anti-H5Nx vaccine. Yang et al. (2022) successfully cloned the M1 gene from the H5N6-Sichuan strain and integrated the H5 and N6 genes into the pFASTBac vector. Using a baculovirus-insect cell expression system, they constructed the VLP. The results demonstrated that 35 days post-immunization with the H5N6 VLP, a significant 2–16-fold increase in neutralizing antibody titers was observed compared to the control group, which was vaccinated with H5 + N6 protein antisera. Additionally, levels of key cytokines such as IL-2, IL-4, IL-5, IFN-*γ*, and TNF were elevated by 2–5 times. These findings suggest that the broad-spectrum H5Nx influenza vaccine, developed with BEVS and utilizing H5N6 VLP as the antigen, effectively induces both humoral and cellular immune responses. However, to date, no clinical studies have been reported for this vaccine.

Unlike HA, NA is less susceptible to antigenic drift, making it an important target for broader protection against multiple influenza strains. Consequently, NA is considered a supplementary antigen to the immunodominant HA in influenza vaccines. Khanefard et al. (2022) genetically modified Sf9 insect cells to stably express the NA gene from the H5N1 influenza virus under the control of the baculovirus IE2 promoter. In the insect cell environment, recombinant NA proteins are synthesized and self-assembled into VLPs. Due to stable expression, NA-VLPs can be continuously produced and released into the culture medium. Immunization of mice with NA-VLPs effectively induced the production of NA-specific antibodies. However, clinical trials for this vaccine candidate have yet to be reported. In another study, Guzman Ruiz et al. (2024) utilized BEVS and the novel virus-free Tnms42 insect cell line to express N2 from A/Kansas/14/2017 (H3N2) on gag-based VLPs. The study compared the efficacy of this VLP formulation to a soluble tetrameric version of the same N2 antigen. Although recombinant protein vaccines often require adjuvants in humans to enhance their immunogenicity, they may also increase reactogenicity. Therefore, vaccine candidates that can function effectively without adjuvants are preferred. Mice vaccinated with 1 μg of N2-VLPs showed significantly better protection against mortality compared to those immunized with the nonadjuvanted soluble N2 form.

The vast majority of existing influenza vaccines are not tailored to address newly emerging influenza B viruses (IBVs). To overcome this limitation, Kang et al. (2022) utilized two distinct IBV strains—B/Washington/02/2019 (B/Victoria lineage) and B/Phuket/3073/2013 (B/Yamagata lineage)—to produce influenza B VLP vaccines using the BEVS. These vaccines were engineered to express HA, NA, or their derivatives. Animal studies demonstrated that mice immunized with the IBV VLPs exhibited a significant reduction in lung viral load and achieved 100% survival when challenged with the Victoria lineage virus (B/Colorado/06/2017). Furthermore, these vaccines conferred cross-protection against infections from mismatched lineage viruses. However, there is currently a lack of clinical trial data to support the clinical efficacy of this approach.

### 3.3 Progress of vaccines in clinical trials and approved vaccines

BEVS-based influenza vaccines have gained approval, with one key example being Sanofi Pasteur’s recombinant quadrivalent influenza vaccine (RIV4), developed by Sanofi Pasteur under the brand names Flublok® and Supemtek® in the EU and Canada (Felberbaum, 2015). This vaccine uses a recombinant baculovirus to express influenza HA genes in Sf900+ insect cells, triggering an immune response that protects against both type A and type B influenza viruses. Clinical trials have shown that Flublok is highly effective, with a study of 9,000 adults over 50 revealing that it reduces the likelihood of influenza by more than 40% compared to other vaccines. Flublok was approved by the FDA in 2013 as the first vaccine capable of targeting both influenza types A and B using BEVS-derived recombinant proteins (Arunachalam et al., 2021).

In 2019, Keshavarz et al. (2019) compared Flublok with two other quadrivalent vaccines: the egg-based Fluzone and the cell culture-based Flucelvax. Their study found that Flublok induced stronger CD4^+^ T-cell and HA-specific antibody responses. Unlike egg-or cell-based systems, which may suffer from structural changes, Flublok avoids antigenic mismatch. Furthermore, recombinant hemagglutinin (rHA) production does not require virus inactivation or chicken embryos, reducing the risk of adverse reactions from impurities like residual egg proteins. The rHA maintains intact epitopes, enhancing its ability to bind and improve vaccine effectiveness.

On November 18, 2020, the European Union approved Sanofi Pasteur’s Flublok Quadrivalent, a recombinant influenza vaccine developed using the BEVS. Flublok offers protection against both influenza A (including H3N2) and B viruses, and it is recommended for adults aged 18 and older (Protein Sciences Corporation, 2016). Two Phase III clinical trials demonstrated that the vaccine’s immunogenicity, safety, and tolerability were comparable to those of egg-based inactivated quadrivalent vaccines in adults aged 18–49. However, in individuals aged 50 and above, Flublok Quadrivalent showed a 30% reduction in influenza incidence, indicating enhanced immunogenicity in this age group compared to traditional vaccines (Dunkle et al., 2017a; Dunkle et al., 2017b).

Additionally, the NanoFlu™ vaccine, developed by Novavax using the SF9 insect cell BEVS, is a quadrivalent rHA protein nanoparticle vaccine (Smith et al., 2017). In Phase III trials conducted in 2019, NanoFlu demonstrated a geometric mean titer (GMT) 24–66% higher and a seroconversion rate (SCR) 11.4–20.4% higher than the Fluzone Quadrivalent vaccine. Following the successful completion of these trials, Novavax submitted an application for FDA approval in 2020.

The safety, immunogenicity, and tolerance of Novavax’s 2009 A/H1N1 VLP vaccine, produced using BEVS, were evaluated through a collaboration between Novavax and Avimex Laboratories in Mexico (López-Macías et al., 2011). The results indicated that the vaccine elicited a strong HAI immune response after a single immunization. Notably, 82–92% of participants achieved protective serum levels (≥40 HAI titer), and 64 to 85% of seronegative individuals reached similar titers. Recent findings suggest that a 15-microgram dose is the optimal and safe choice for individuals aged 18–64 years. Furthermore, protective antibody levels were maintained for up to 2 years post-vaccination, underscoring the vaccine’s strong safety profile, immunogenicity, and long-lasting efficacy.

These studies underscore the potential of BEVS in the development of influenza vaccines, providing a solid foundation for future exploration of both seasonal and pandemic influenza vaccine candidates (Valero-Pacheco et al., 2016).

## 4 Respiratory syncytial virus infection

### 4.1 Epidemiology of respiratory syncytial virus infection

RSV infection is a leading cause of neonatal mortality, responsible for up to 2.3% of global deaths in neonates (0–27 days) and approximately 6.7% in infants aged 28–364 days. In children aged 1–4 years, RSV contributes to 1.6% of mortality (Shi et al., 2017). In adults aged 50 and older, the mortality rate among hospitalized patients with RSV infection ranges from 6 to 8% (Colosia et al., 2017). China has one of the highest global incidences of RSV, with annual hospitalizations for children under 5 years of age ranging from 619,000 to 948,000, including between 215,000 and 500,000 infants (Hall et al., 2009; McLaughlin et al., 2022). In the United States, RSV hospitalization rates for children under five range from 58,000 to 80,000, while for adults aged 65 and older, the rates range from 60,000 to 160,000 (McLaughlin et al., 2022; Zheng et al., 2022; Havers et al., 2022). These statistics underscore the urgent need for an effective RSV vaccine.

RSV is an RNA virus with a negative-sense genome encoding several proteins, including the nucleocapsid (N), envelope (E), fusion (F), attachment (G), membrane (M), small hydrophobic (SH), and large polymerase (L) proteins. The F protein plays a critical role in viral fusion with host cells, while the G protein facilitates viral attachment. The development of vaccines against RSV has focused on six major approaches: recombinant vector vaccines, subunit vaccines, particle vaccines, attenuated live vaccines, chimeric vaccines, and DNA vaccines. Baculovirus vector vaccines offer significant advantages in terms of safety by reducing the pathogenicity of the vector virus, making them particularly suitable for protecting vulnerable populations, such as infants and the elderly, from RSV (Mazur et al., 2023). Notably, the F and G proteins are key targets for inducing neutralizing antibodies, as they are involved in the virus’s adsorption and fusion processes, making them critical for eliciting strong antiviral responses. Research into RSV vaccines based on baculovirus vectors often emphasizes these surface proteins.

### 4.2 Preclinical investigations

Previous studies have demonstrated the efficacy of baculovirus-expressed RSV vaccines in eliciting robust immune responses against RSV (Olmsted et al., 1986; Kohlmann et al., 2009; Johnson and Collins, 1988). Notably, the F glycoprotein of RSV has been shown to induce potent neutralizing antibodies and RSV-specific cellular immunity, offering significant protection and cross-protection against a broad range of RSV strains. Blanco et al. (2014) described a candidate vaccine utilizing anchorless RSV F protein expressed in insect larvae infected with baculovirus. This vaccine, which incorporates monophosphoryl lipid A (MPL)—a mild agonist of toll-like receptor 4 — was administered through nasal priming followed by intradermal booster injections. The results demonstrated that intranasal vaccination with the recombinant F protein vaccine conferred partial pulmonary protection against subsequent RSV infection, leading to reduced lung injury compared to controls. The adjuvanted F protein vaccine further enhanced pulmonary protection, significantly mitigating tissue damage. Despite these promising findings, it is important to note that this research has not yet progressed to clinical trial stages.

Luo et al. (2022) explored the potential of pre-fusion conformation virus-like particles (pre-F VLPs) as a promising candidate for an RSV vaccine. By designing RSV F protein mutants to stabilize the pre-F conformation and using BEVS to generate VLPs incorporating the pre-F protein, they demonstrated that immunizing mice with pre-F VLPs resulted in the upregulation of IFN-*γ*, IL-2, and IL-10, alongside a downregulation of IL-4 and IL-5. These findings suggest that pre-F VLP immunization effectively induces the production of RSV-neutralizing antibodies and elicits strong immune responses, positioning pre-F VLPs as a promising vaccine candidate for RSV.

Similarly, Lee et al. (2023) used BEVS to generate VLPs expressing the pre-F protein, G protein, and a combination of both (pre-F + G) antigens of RSV. In murine models, these VLPs demonstrated significant efficacy in mitigating RSV-induced eosinophil infiltration and lung inflammation. The pre-F + G VLP vaccine notably reduced viral titers and lung inflammation, while increasing the proportion and count of CD4^+^ T cells. The inclusion of multiple antigens in the VLP vaccine enhanced its protective efficacy, suggesting that combination antigen vaccines could offer optimal protection against RSV.

### 4.3 Progress of vaccines in clinical trials and approved vaccines

Currently, no baculovirus vector vaccines for RSV infection have received approval. However, vaccines utilizing baculovirus as a vector have advanced to Phase III clinical trials. In 2012, Novavax utilized the BEVS to express the Fusion (F) glycoprotein, which is located on the surface of the RSV envelope, and developed it into a vaccine candidate (Glenn et al., 2013; Smith et al., 2012). The Phase III trial results indicated that the vaccine achieved an efficacy of 39.4% in preventing RSV-related lower respiratory tract infections among infants aged 90 days, which fell short of the 50% efficacy threshold recommended by the World Health Organization (Madhi et al., 2020). Moreover, the vaccine demonstrated a negative efficacy of-7.9% in preventing moderate to severe RSV-related lower respiratory tract infections in individuals aged 60 and above (ClinicalTrials, 2016). Consequently, the baculovirus vector vaccine for RSV developed by Novavax was deemed unsuccessful, as the Phase III trial outcomes did not meet the necessary efficacy standards.

Although no baculovirus vector vaccines for RSV have been approved by the FDA, non-baculovirus vector vaccines have gained approval. Pfizer’s Abrysvo, based on the pre-F protein of RSV, was approved in May 2023 (Abrysvo, 2023; Syed, 2023) and GSK’s Arexvy, combining pre-F with the AS01E adjuvant, followed in July 2023 (Arexvy, 2023). Bavarian Nordic’s MVA-BN RSV vaccine, developed in 2017, completed Phase III trials and received Breakthrough Therapy Designation (BTD) from the FDA in February 2022. While MVA-BN RSV uses a vaccinia virus vector similar to baculovirus, constructing vaccinia vectors is more complex and slower, making baculovirus vectors more efficient for vaccine development. Although baculovirus vector vaccines for RSV have not yet succeeded in Phase III trials, they hold strong potential for improved safety and efficacy. Baculovirus vector vaccines remain a promising avenue for future research.

## 5 Middle East respiratory syndrome

### 5.1 Epidemiology of Middle East respiratory syndrome

MERS-CoV, the first known lineage B coronavirus to infect humans, has caused 2,608 laboratory-confirmed cases globally between April 2012 and October 2023, with the majority (approximately 2,199) occurring in Saudi Arabia and 857 associated fatalities (MERS Situation Update, 2023; Banik et al., 2015). The MERS-CoV genome encodes four structural proteins: spike (S), envelope (E), membrane (M), and nucleocapsid (N) proteins. The E protein plays a critical role in viral assembly, intracellular transport, and budding, and its absence results in viral attenuation (DeDiego et al., 2007). The S protein facilitates virus binding to host cells, while the M protein shapes the viral particle and determines its morphology. The N protein binds to the viral RNA genome to form the nucleocapsid. Together, these proteins coordinate the adherence, entry, and replication of MERS-CoV within host cells. Due to its crucial role in viral entry, the S protein is a key target for MERS-CoV vaccine development. Recombinant expression of the S protein using BEVS has proven effective in eliciting immune responses that confer protective immunity against MERS-CoV (Mubarak et al., 2019).

### 5.2 Preclinical investigations

The expression of the MERS-CoV E protein was successfully achieved using the BEVS, as demonstrated by Gao et al. (2013), Chen et al. (2011), Yousefi et al. (2012), and Ahrens et al. (2000) as a membrane protein present at low levels only in viral particles, studying the E protein has been challenging (Kitts et al., 1990; Alsaadi et al., 2023). However, BEVS has enabled the exploration of the E protein’s biological functions, advancing MERS-CoV vaccine development. The spike protein (S) is a key target in most ongoing MERS-CoV vaccine efforts. Composed of two subunits, S1 and S2, RBD is part of the S1 subunit. Kovalenko et al. (2023) and Lan et al. (2015) developed a candidate vaccine based on the MERS-CoV RBD using the BEVS platform. This vaccine, combined with an adjuvant, induced strong T-cell-mediated immune responses and elevated levels of neutralizing antibodies in rhesus macaques. Furthermore, the vaccine effectively reduced viral loads in the lungs, trachea, and oropharyngeal region, alleviating pneumonia progression during MERS-CoV infection.

To enhance vaccine efficacy, Jung et al. (2022) developed a subunit vaccine using genetic engineering in Chinese hamster ovary (CHO) cells. They fused the S1 subunit of the MERS-CoV spike protein with the human IgG_4_ Fc fragment to improve the antigen’s pharmacokinetics and boost its immunogenicity. To further enhance the immune response, they added a gelatin adjuvant. In a mouse trial, the vaccine with the adjuvant-finduced higher levels of neutralizing antibodies compared to the vaccine without it. After 7 days, mice receiving the adjuvanted vaccine showed normal lung tissue, while those without the adjuvant had signs of inflammation and edema. These findings suggest that the subunit vaccine with gelatin adjuvant is a promising candidate for preventing MERS-CoV infection.

### 5.3 Progress of vaccines in clinical trials and approved vaccines

As of now, no vaccine for MERS-CoV has been approved. Consequently, developing effective vaccines to control its spread and reduce the risk of a pandemic is of utmost importance. Several candidate vaccines are currently under development, including spike (S) protein nanoparticles (Coleman et al., 2014), modified vaccinia virus vectors (Song et al., 2013), full-length S DNA-based platforms, and immunogens based on the S1 subunit protein (Wang et al., 2015). These vaccines have all demonstrated the ability to induce neutralizing antibodies against MERS-CoV. Among them, VLPs, which are pure protein subunit vaccines that mimic the natural structure of viruses, offer significant promise. VLPs not only induce strong humoral and cellular immune responses but also carry a lower risk of virulence reversion compared to attenuated or inactivated vaccines (Noad and Roy, 2003; Rodríguez-Limas et al., 2013). Moreover, they can be produced in biosafety level 2 (BSL-2) facilities, ensuring high safety standards. Given the success of baculovirus vector vaccines in the development of SARS-CoV-2 vaccines, we believe that leveraging baculovirus for recombinant VLPs holds great potential and feasibility for developing an effective MERS-CoV vaccine.

## 6 Conclusion

Respiratory infectious diseases, caused by viruses such as SARS-CoV-2, influenza, RSV, and MERS-CoV, pose significant global health risks. These viruses are responsible for widespread outbreaks and severe disease. Vaccines have emerged as a key tool in preventing these infections. However, traditional vaccine development faces challenges, including long production cycles, complex procedures, and numerous influencing factors. In contrast, baculovirus vector vaccines offer notable advantages, including enhanced flexibility and safety, making them a promising alternative for vaccine production.

Baculovirus vector vaccines are typically produced using the BEVS. This process begins with the transformation of a plasmid containing the target gene into *Escherichia coli* containing the baculovirus plasmid. Homologous recombination between the transfer and baculovirus plasmids generates recombinant baculovirus DNA, which is then used to infect insect cells. The resulting recombinant proteins are collected and purified to create the vaccine. BEVS has been successfully employed in the development of vaccines targeting respiratory pathogens, such as the S-RBD gene for COVID-19, the HA or NA genes for influenza, and the F protein for RSV. Additionally, the S-RBD gene has also been utilized in MERS-CoV vaccine development.

This review summarizes the progress of baculovirus vector-based vaccines for respiratory diseases, highlighting preclinical and clinical trials, as well as approved vaccines. Our goal is to provide a comprehensive overview of the potential of BEVS in the development of vaccines for these prevalent infections, providing some theoretical basis for subsequent innovative clinical studies. In this paper, we only discusses baculovirus vector vaccines related to respiratory diseases, favoring a review, summary, and comparison of existing studies. Additionally, we have not conducted an in-depth systematic study on the differences in the performance of baculovirus vector vaccines across various populations, such as children, the elderly, and immunocompromised individuals. The immune response may be different in these populations, and further studies are needed to optimize vaccine design. Although baculovirus vector vaccines are considered to have a high safety profile, this review does not provide a detailed discussion on potential risks associated with their long-term use, such as immune tolerance or cross-reactivity. Future research could focus on: (1) Combination Vaccines Design: few baculovirus vector combination vaccines exist, but high-capacity gene insertion capability of baculovirus offers significant potential for future development. (2) Enhanced Immunogenicity: The relatively low immunogenicity of baculovirus-vectored vaccines necessitates enhancement strategies, such as the design of suitable adjuvants. (3) Investigation of long-term immune efficacy: Data on the long-term immune efficacy of baculovirus vector vaccines are limited, particularly regarding the durability of immune responses and the necessity for booster doses, and more in-depth studies are needed in this area.
